# Qualitative participatory needs assessment in long-term care facilities: groundwork for a workplace health promotion program based on traditional, complementary and integrative medicine (TCIM)

**DOI:** 10.3389/fmed.2025.1671029

**Published:** 2025-12-05

**Authors:** Fabian Voigt, Judith Czakert, Wiebke Stritter, Sophia Shir Teng Yap, Georg Seifert, Christian S. Kessler

**Affiliations:** 1 Charité - Universitätsmedizin Berlin, Corporate Member of Freie Universität Berlin and Humbold-Universität zu Berlin, Charité Competence Center for Traditional and Integrative Medicine (CCCTIM), Berlin, Germany; 2 University of São Paulo, Faculty of Medicine, São Paulo, Brazil; 3 Immanuel Hospital Berlin, Department of Internal and Nature-Based Therapies, Berlin, Germany

**Keywords:** workplace health promotion, traditional, complementary and integrative medicine, needs assessment, nursing, long-term care facility, qualitative research, healthcare professionals

## Abstract

**Background:**

The workload for nursing staff in long-term care facilities will increase in the coming decades, making initiatives to promote health in the work environment increasingly important. The pilot project “Healthy Care Cares for Health” (HCCH) aims to develop, implement, and evaluate a TCIM-based program for workplace health promotion (WHP) in long-term care facilities. To create an overview of health-related challenges and resources of the pilot facility, a needs assessment (NA) with participatory elements was conducted to include participant input.

**Methods:**

The NA was based on the framework of a quality-oriented Rapid Participatory Appraisal (RPA). Data was collected through semi-structured interviews and participant observation. The interview sampling used maximum variation based on participant socio-economic data including occupation, age, and gender (*n* = 19). The data was analyzed using deductive-inductive qualitative content analysis. The results from the interviews and observations were contrasted, integrated, and validated in communication with the target group.

**Results:**

The NA revealed a complex interplay of health-related resources and challenges. Key challenges included stressors (e.g., insufficient breaks), physical and psychological strain (e.g., emotional stress, physical workload) and unhealthy lifestyle habits, particularly related to eating and exercise. At the same time, staff members demonstrated valuable individual resources and strategies (e.g., trust, a sense of coherence). They also showed approaches related to safety, stress management, workplace relationships, nutrition and physical activity. Notably, some participants already make regular use of TCIM strategies (e.g., breathing exercises).

**Discussion:**

The RPA-based NA is an effective, resource-efficient way of exploring target group- and setting-specific data on health-related challenges and potential in long-term care facilities. These results form a crucial basis for a context-adapted WHP program, with TCIM-related resources and strategies guiding the development and implementation of the planned TCIM-based WHP. TCIM approaches offer sustainable, low-threshold, and resource-efficient health promotion measures that can enhance staff health, care quality, and patient safety in long-term care and are also relevant for primary health care professionals broadly. Furthermore, the NA, with its participatory elements based on RPA recommendations, supports continuous target group involvement and provides a basis for consistent nursing staff participation in a WHP program.

## Introduction

Around 1.7 million nursing staff work in Germany, more than half a million of them in long-term care facilities (1). Research results have shown that both objective workload and subjective stress levels in the care sector are above average compared to many other occupational groups (2). Nursing staff in long-term care facilities in particular are more frequently exposed to psychological and physical demands [e.g., high stress levels, violence experiences, high physical workload (2–4)] than employees in other areas of nursing care (2, 5–7). The potential health consequences of these stressors (include burnout, depression or musculoskeletal disorders (3, 5, 8–10)) lead to above-average absences compared to other occupational groups (5, 11). These demands are exacerbated by the shortage of skilled workers in the healthcare sector (12), which has existed for years and has resulted in intensified work and increased time pressure for existing staff (13, 14). Predicted demographic changes will lead to an increase need for skilled workers in healthcare services in Germany (ex.: 15, 16). It can be concluded from this that the health, job satisfaction and well-being of nursing staff in long-term care facilities will be under even greater strain in the future. Many nursing staff already see their long-term ability to work at risk and doubt that they will be able to work until retirement under current conditions (2). Against this backdrop, workplace health promotion (WHP) measures are becoming increasingly important to strengthen the health and ability to work of nursing staff in general and long-term care facility staff in particular (7, 17). Although this study focuses on the setting of long-term care, it could also be interesting in the future to specifically strengthen the health of nursing staff in primary health care, as this group also plays an essential role in the overall health care system.

As part of the pilot project *“Healthy Care Cares for Health” (HCCH)* this need is to be addressed through the conceptualization, implementation and evaluation of a TCIM-based, participatory and multimodal WHP program for nursing staff in a long-term care facility. Currently, there are hardly any programs for this target group that integrate the potential of TCIM approaches with multimodality and participation in a single program (18). The integration of evidence-based TCIM methods – such as mindfulness, breathing exercises, yoga or aromatherapy (19, 20) – offers promising prospects in this context. These procedures are health-promoting, holistic and resource-oriented, and can also be individually adapted to the needs of the staff and their work context (21). This perspective is further reinforced by the WHO’s recommendation for their integration into WHP (22). In addition, good adaptation promises high adherence and sustainability (21). In long-term care facilities, studies show that a high acceptance of TCIM-based approaches have positive effects on health, well-being and the relationship between staff and residents (23, 24). Further studies show that mind–body medicine techniques can help improve burnout, stress, depression, and job satisfaction (ex. 25–28). Recent data on the use of TCIM measures in Germany indicate that a notable proportion of nurses may already be familiar with TCIM practices (29). The aim of the pilot project is to enable the employees of the pilot facility to develop resource-oriented health behavior in their work context, implement appropriate measures and integrate them sustainably into their daily work routine.

The development of a TCIM-based, participatory and multimodal WHP program, that considers the needs and resources of the nursing staff as well as the setting-specific framework conditions, requires in-depth knowledge of the health-related circumstances of the pilot facility and the characteristics of the staff working there. A comprehensive needs, requirements and setting analysis in the context of a “needs assessment” (30) is therefore a basis for an appropriately adapted WHP program. Based on the findings of the 2023 scoping review “*We cannot just run a standard program*” (18), which served as a preliminary study for the “*HCCH”* pilot project, key prerequisites and success factors for effective WHP in long-term care facilities were identified. These include the importance of active participation of the target group in all phases of program development and implementation. This aspect is therefore explicitly addressed in the needs assessment. The participatory approach of a qualitative Rapid Participatory Appraisal (RPA) (31, 32) serves the methodological framework, and is particularly suitable for time-efficient but at the same time in-depth participatory surveys.

The research objectives of the needs assessment was (a) to gain a comprehensive understanding of the specific health challenges, existing resources and contextual conditions of the nursing staff in the selected long-term care facility. The focus was on the specific framework conditions of the entire pilot facility as well as the everyday work routine and experience of the staff within this setting. At the same time, the analysis should (b) provide information on the extent to which the participatory elements used in the RPA can contribute to creating a needs-oriented and practical basis for subsequent program design. Against this background, the following qualitative research questions were asked as part of the needs assessment:

- *What health-related barriers, challenges and obstacles do the employees of the pilot facility encounter in their day-to-day work?*
- *What health-related resources relating to stress, nutrition and exercise are available to the employees and how are they used?*
- *What are the health-promoting and health-inhibiting features of the entire pilot facility?*
- *To what extent can the Rapid Participatory Appraisal approach contribute to the successful participation of the target group as part of the needs assessment?*

## Methods

The needs assessment is based on a qualitative-descriptive approach (33) and is oriented toward the concept of a Rapid Participatory Appraisal (RPA) (31, 32). An RPA enables information collection, analysis and prioritization with the involvement of the target group in order to identify health-relevant factors from their perspective and take into account contextual conditions (30). Within this resource-saving approach, relevant information on the health, behavior and attitudes of the target group can be collected within a short period of time in order to use it for the development of further measures (32). The RPA follows three principles: 1. relevance; 2. adaptation of the survey to the existing conditions and situations; 3. inclusion of the target population (32). The recommendations for RPA for 1. planning and 2. data collection and analysis by Pepall et al. (32) were considered as a framework for conducting the needs assessment.

### Ethics and Quality

A positive ethics vote (EA4/251/23) was issued on May 29, 2024 for the implementation of the needs assessment. The presentation of the methodological procedure was based on the “Standards for Reporting Qualitative Research” (SRQR) (34).

### Design

A qualitative method triangulation was carried out for the health-related needs assessment in the pilot facility, as recommended by Pepall et al. (32) in the context of an RPA. The basis of the between-methods data triangulation (35) was formed by three data sources that enable different perspectives on the research questions: Interview data from the target group, observations in the in-house setting and structural data from the institution. The predominantly qualitative approaches allow the recording of any, even unexpected, content that could be relevant within the setting. The overview of the circumstances and specifics of the setting was further supplemented by the inclusion of facility-related information in the form of structural data. The different data corpora were contrasted and integrated in the analysis in order to obtain a comprehensive overall picture of the challenges and support factors for the health of the staff at the pilot facility (35).

### Participation

The participatory approach of the RPA was implemented in the needs assessment as follows: The commitment to support the needs assessment and the entire WHP project on the part of the company management was obtained in advance. The management team and employee council of the pilot facility were involved in the concept from the outset through regular exchanges. The implementation and feasibility of the needs assessment were jointly coordinated. From the onset, all staff were informed about the key steps of the project during several face-to-face meetings held during working hours as part of regular staff meetings. All meetings were conducted in accordance with the principles of ‘participation’ (e.g., encouraging discussion, questions and answers; exchange; communicative validation of results) and’information exchange’ (e.g., providing information about the pilot project, the needs assessment and the next steps). In addition, emails were regularly sent via the institution’s internal mailing list to provide information on the current status and next steps of the project. They were also used to thank participants for their participation in the data collection. The research team encouraged openness in exchange to answer questions from the onset.

### Data collection

To gather information on the work experience of staff and health-related characteristics of the pilot facility, the following measures were carried out in July and August 2024: (a) semi-structured, guideline-based interviews with facility staff and (b) protocol-based observations. In addition, structural data (e.g., number of employees, job titles, number of residents) about the pilot facility was collected. All participants were fully informed in advance and signed a declaration of consent.

#### Interviews: sampling and implementation

The focus of the guideline-based interviews was on the topic of health in everyday working life and followed the criteria of openness, specificity, contextuality, and relevance (36). The guideline allows the principle of moving from the general to the specific (36). Accordingly, the interview begins with an open narrative stimulus on the topic of everyday work in the pilot facility. This is followed by more specific questions on the topics of ‘structures and tasks’, ‘work experience’ and ‘health in everyday work’, which either build on what has already been said or introduce new content. The flexible structure of the guideline allows for the coverage of a wide range of health-related topics and enables appropriate response to the conversation (37). The interview guideline was validated in advance as part of a pre-test to check the comprehensibility, logical sequence and relevance of the questions. The feedback provided allowed for optimizations to be made to the guideline before the data collection began.

All staff members of the pilot facility (*n* = 35) were eligible to be included in the data. A targeted strategy of maximum contrast (37, 38) based on the characteristics of function, occupational group, age, and gender was chosen for the compilation of the sampling. The aim was to include as wide a range of variations and perspectives as possible with regard to the topics of interest (38). The number of interviews was planned in advance as n = 10–20, depending on the occurrence of a thematic saturation effect (39). Theoretical saturation was determined through a parallel and iterative process of data collection and analysis. It was achieved when no new significant findings, codes, themes, or categories could be extracted from the data and the statements become increasingly repetitive (39). Access to the target group had already been established through previous informational events on the study. Selected employees were personally asked to participate. The time and place of the interviews were agreed individually with all participants. The two researchers (FV, JC) took care to adapt to the conditions of regular work processes in the pilot facility in order to avoid disruptions. In concrete terms, this meant that some interviews were interrupted, took place while walking or during work, or that a quiet room had to be found together with the interviewees at the agreed time. All interviews took place during working hours and were recorded on audio media.

#### Participant observation

The focus of the participant observations, carried out by two researchers (FV, JC), was on health-related resources and challenges as well as processes and routines in everyday work. The observations focused on accompanying individuals who had already been interviewed in order to supplement their statements with observations. In addition, individuals who had not been interviewed were accompanied in order to gain insights into their everyday work. Moreover, observations were made of specific situations and processes that emerged from interviews that are particularly insightful for health-related topics. The observation protocol developed in advance provided guidance for orientation during observations (36, pgs 49–51). The participatory nature of the observations consisted of accompanying staff in their respective work processes and occasional *ad hoc* discussions (e.g., questions about work processes, responding to offers of communication, initiating discussions on health-related observations) (38, pgs 357-359).

### Focus group discussion

After analyzing the individual interviews, a focus group discussion (38) was held with a large number of employees as part of a staff meeting. The focus of the moderated session was provided by the presentation of the illustrated key findings in poster format (see Supplementary Appendix 1). Feedback of the results to the target group once again allowed staff to participate, enabling them to contribute their associations, opinions, and thoughts on the results. In addition to communicating the validity of the results (37), the focus group discussion also provided an opportunity to deepen and expand on the findings presented. The core findings of the meeting were then discussed and recorded by the research team (FV, JC) (see chapter “Results”).

### Data analysis

The interviews were automatically transcribed using AI-supported software called ‘f4’ and then manually revised. Semantic adjustments were made to the content, and spelling and grammatical corrections were made without altering the original meaning. The revised transcripts were entered into the computer-assisted analysis software ‘MAXQDA24’. The analysis process - carried out in parallel by FV and JC - was based on a deductive-inductive qualitative content analysis (40, 41). A pre-developed (deductive) code tree of main categories taken from the research questions laid the foundation for the further analysis process. Regular discussions (FV, JC) about the newly developed inductive categories generated from the data and the code tree led to several adjustments to the category systems. Definitions and structures were continuously adapted. The categories were discussed in several joint meetings, correlated, and summarized graphically. The summary was reviewed and discussed by an extended team of qualitative researchers. Regular reflection and discussion of the analysis steps and results with qualitative researchers can be seen as a second way of communicative validation of the results through researcher triangulation. This is considered a criterion of quality for qualitative analyses (35, 42).

The core findings were discussed with an illustrator and translated into graphics. The graphic design was developed in several rounds of consultation with the research team (FV, JC) to ensure that the results were translated into illustrations without any loss in content. The final illustration (see Supplementary Appendix 1) was used as the focus for the focus group session on communicative validation (see also: “Results from the focus group session” in Supplementary material). This corresponds to the final report of the key findings for stakeholders recommended within the scope of an RPA (32).

## Results

To identify health-related resources and challenges in the pilot facility, a total of 19 employees were interviewed by two researchers (FV, JC) as part of the needs assessment in July and August 2024. In addition, participant observation was conducted in eleven days in the course of everyday work. A detailed description of the characteristics of the interview sampling can be found in the appendix (see Supplementary Appendix 2).

The data was analyzed deductively through main categories based on the central research questions. These main categories - work environment, working conditions, resources, strategies, health challenges, and wishes for WHP - could be assigned to two overarching themes: extrinsic factors (conditions of the workplace setting) and intrinsic factors (personal perspectives of the nursing staff). In the further course of the analysis, 57 subcategories were inductively derived from the data material, which further differentiated the main categories (compare this with the procedure for thematically structured qualitative content analysis within data evaluation in chapter “Methods”). This approach made it possible to uncover a multifaceted picture of the interrelated health-related factors within the pilot facility from the perspective of the staff. At the organizational level, the work environment and working conditions encompass both the perception of potentials and stressors. At the individual level, in addition to physical and psychological stressors and unhealthy eating and exercise habits, resources and personal strategies in these same areas were also identified. The complete category system with sample interview quotes is shown in Table 1. A comprehensive description of all inductively developed categories, as well as the findings from the participant observations and the focus group discussion, can be found in Supplementary material. In view of the TCIM-based concept of the planned WHP program, the strategies identified among the staff (intrinsic factors) already showed signs of approaches that can be attributed to TCIM. These strategies include mindfulness toward oneself and others, breathing exercises, and experiences of nature and art.

**Table 1:** Overview of the complete category system with exemplary interview quotes.

Main category (deductive) and subcategory (inductive)	Exemplary quote
*Work environment (extrinsic factors)* *Potentials:* Aids: Promotion of healthy exercise and relaxation (1) Aids: Promotion of healthy nutrition (2) Spatial conditions (3) *Challenges:* Physical strain	*“Yes, of course we also have many aids, such as mobility aids, devices, and lifts that we can use. […] A sliding mat or something similar that I can use to move residents without straining my back.”* (P_06) *“Well, our management always puts a fruit bowl out for us. Once a week, and by the end of the week it’s always full again.”* (P_19) *“Yes, exactly. Outside, when you are sitting outside in the garden or on the terrace, it’s so nice.”* (P_06)
*Working conditions (extrinsic factors)* *Potentials:* Good communication Social relationships (1) Appreciation & recognition (2) Work organization Breaks *Challenges:* Interpersonal conflicts Communication problems Shift work (3) Understaffing (4) Work intensification Work interruptions Lack of breaks (5) Poor work organization	*“That’s why I like coming here. Everyone looks out for each other. Yes, and that’s good.”* (P_04) *“Yes, at the end of the day, you get a smile from older people. And when you have not been there for a few days: Oh, great, you are finally back.”* (P_14) *“There’s always this pattern of working Monday to Friday, one day off, then four days again, one day off, then four days again […] You’re exhausted. You feel like all you do is work.”* (P_03) *“So, there’s already time pressure and staff shortages […]. And that really puts pressure on me, those are the stressors that are weighing on me right now.”* (P_03) *“But sometimes it really happens: I decide that now I’m going to take a break. And then someone comes along, calls, or you have to go somewhere quickly, or someone wants to talk to you.”* (P_09)
*Resources (intrinsic factors)* Trust and security Positive attitude Sense of coherence (1) Intrinsic motivation Awareness of (un)healthy exercise Awareness of (un)healthy nutrition (2)	*“The thing is, I was born to be a nurse. I love my job.”* (P_19) *“And I just realized that it’s not good for me when I sit upstairs next to the computer and eat something quickly on the side. That’s why I now go downstairs regularly and take my half-hour break here in peace.”* (P_05)
*Strategies (intrinsic factors)* Habituation and acceptance Mutual support Flexibility Establishing structure and priorities Recognizing boundaries and limits Experience and decision-making skills Leaving challenging situations (1) Maintaining a calm attitude Finding of meaning (create a sense of coherence) Rational behavior Physical relaxation Spending time in nature (2) Search for peace and solitude (3) Breathing exercises (4) Music Communication and coordination (5) Attention and mindfulness Strengthening relationships and teamwork Openness, respect, appreciation (6) Healthy eating habits Healthy exercise habits	*“And then I have to leave the situation and say: I’m just going to go away for a while. It’s much better when you are more relaxed again. And then I can deal with the problems better.”* (P_09) *“I grab my bottle and sit outside for five minutes in the fresh air. After that, I feel much better.”* (P_05) *“I need peace and quiet. There are also phases when I really do not want to talk at all, but just want to be quiet. Peace and quiet […] Just peace and quiet. Nothing else.”* (P_04) *“Then I sit outside for five minutes. Yes, take a deep breath, breathe in and out deeply, very calmly. And then I can go back upstairs feeling deeply relaxed. That’s good.”* (P_05) *“You talk about it, bring it up at staff meetings, or take colleagues aside and say: You did not do that right, maybe you could do it better.”* (P_02) *“Yes, I would simply call it intercultural cooperation, working with people from different countries […]. Everyone is different. You have to be open to that.”* (P_03)
*Health challenges (intrinsic factors)* Physical factors Emotional strain (1) Lack of expertise and experience Lack of motivation Lack of empathy Inability to set boundaries (2) Loss of control Unhealthy eating habits Unhealthy exercise habits (3)	*“For me, death is something completely normal. It’s part of life. And for me, it’s only really difficult when someone dies a really painful death. When someone has to suffer for weeks. That can sometimes be very stressful.”* (P_05) *“I’m still not very good at setting boundaries […]. Somehow, I still feel like I want to make sure that everyone is well cared for.”* (P_15) *“But as I said, this physical aspect, whether it’s sitting or walking too much, is very stressful […] Sitting for too long is not good, and running around for too long is not good either.”* (P_03)
*Wishes for WHP (intrinsic factors)* Sports and relaxation offerings (1) Improvement of the working atmosphere (2) Improvement of workplace conditions Improvement of work structure	*“Or maybe 30 min of meditation training. Something you can really do in a small group. That would be great.”* (P_05) *“Because at my previous employer, we had team days for team building […] Yes, you had to come together as a team over the years. And you saw that it worked.”* (P_03)

P, participant.

**Figure 1:**
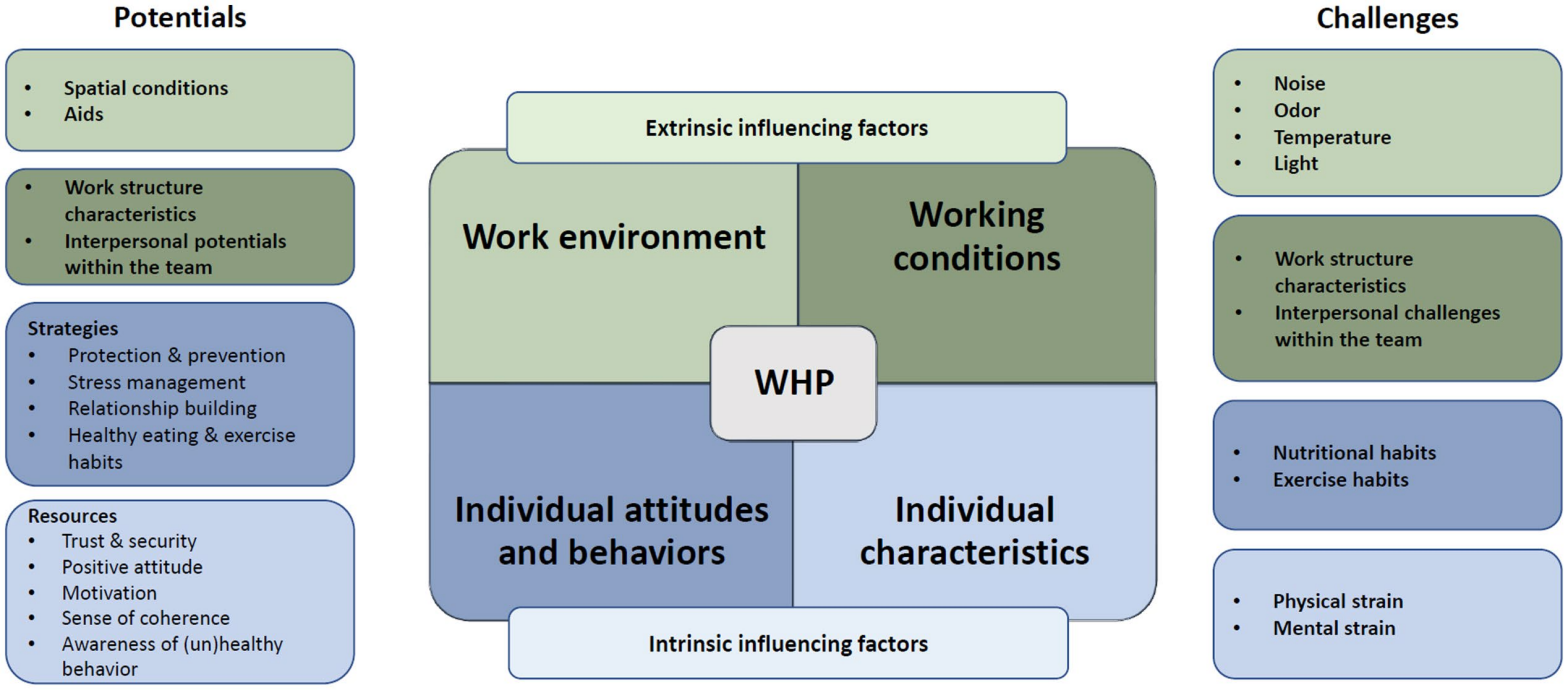
Potentials and challenges of extrinsic and intrinsic factors influencing health in relation to workplace health promotion (WHP).

A supplementary, overarching presentation of the results of the needs assessment summarizes the potentials and challenges of extrinsic and intrinsic factors influencing health in the context of WHP (see Figure 1).

The division into extrinsic and intrinsic influencing factors allows a distinction to be made between factors that (can) affect WHP at the organizational level and at the individual level. From this, measures can be derived that target the nursing staff themselves or make their environment more health-promoting. Specific potentials and challenges can be identified in the categories of *work environment* and *working conditions* (extrinsic influencing factors) as well as in the categories of *individual attitudes and behaviors* and *individual characteristics* (intrinsic influencing factors). This allows the further “*HCCH”* implementation process to consider the specific conditions of the pilot facility and the needs of the staff.

## Discussion

Considering the specific working conditions of nursing staff and the associated health challenges and potentials is considered a key aspect in the development of targeted WHP measures in the nursing sector (43). A needs assessment (30) based on an RPA (31, 32) was conducted as part of the “*HCCH”* pilot project to explore the health challenges and resources of staff in the selected pilot facility. Given the limited time available, the RPA approach proved particularly suitable for involving staff at a low level and establishing them as an integral part of the process right from the start. The comprehensive results of this study indicate that a qualitative-oriented needs assessment incorporating the principles and recommendations of an RPA is an effective tool for identifying health-related factors in the work environment, working conditions and individual attitudes, behaviors and characteristics of the target group within the pilot facility. For the sustainable implementation of WHP programs, it is essential to adapt them to the existing needs and conditions within a specific setting. A needs assessment that incorporates the perspectives, experiences and opinions of the target group provides the necessary information for this. The integration of qualitative research methods is therefore recommended for future research and WHP intervention projects.

In view of the intrinsic and extrinsic health challenges of the target group, similarities in content with relevant research findings in the setting of long-term care facilities can be identified. These research findings document similar stress factors such as shift work, understaffing, lack of breaks, stress and work pressure due to work intensification, work interruptions, emotional strain and interpersonal conflicts (2, 5, 6, 44). In addition, unhealthy movement patterns such as lifting and carrying heavy loads (5, 6, 45) and unhealthy eating habits (46) are described. The health resources of the staff in the pilot facility identified in the needs assessment prove to be only partially comparable with other results, as the resource-oriented perspective in the field of WHP in long-term care facilities is rarely the focus of research. In the few relevant studies, particularly the aspects of “social relationships” and ‘mutual support’ (5, 13) as well as the “existence of meaningfulness” (e.g., the feeling of making an important contribution to society) (2, 5, 6) emerge. These resources also represent significant factors for health stability and well-being of our target group in long-term care facilities. Given the existing research gap on resources and health-promoting strategies for nursing staff, particularly in long-term care facilities, the results offer valuable starting points for future research projects, which we believe are urgently needed.

By aligning the needs assessment with the principles and recommendations of an RPA, the results enabled key topics to be identified that are of particular importance for the development and implementation of further WHP projects. They will be examined in greater depth and discussed below: (a) Participation in WHP: needs assessment as an opportunity for actively involving the target group, (b) Supportive management culture: the importance of company management for needs assessment in WHP programs, (c) Focus on TCIM: use of existing TCIM-based resources and strategies for WHP, (d) Relevance of TCIM-based WHP programs for the primary health care sector.

### Participation in WHP: needs assessment as an opportunity for actively involving the target group

In health promotion, active involvement and participation of the target group is highlighted as a decisive prerequisite and promoting factor for successful WHP programs (47–51). Participation is central to health promotion, as it enables the target group to be continuously involved in decisions that affect their health. Active involvement in the development, implementation and evaluation of health promotion interventions increases their acceptance and effectiveness, as they can be better tailored to the needs of nursing staff (47, 49, 50, 52). In general, sustainable implementation of WHP programs is more likely if employees identify with the program and are intrinsically motivated to participate in it (51). When implementing a sustainable WHP program, it is essential to consider the specific health-related needs, resources, and challenges of nursing staff. However, nursing staff is generally considered to be reluctant to openly express individual opinions about their work environment to their superiors (53). This underscores the need for appropriate participatory procedures that can be used to highlight healthcare issues at the organizational level. This need was specifically addressed in the needs assessment and is also reflected in the results, whereby the orientation toward an RPA (see chapter ‘Methods’) contributed significantly to the success of the target group’s participation. The needs assessment enabled the target group to be involved through the systematic collection and analysis of individual opinions, perceptions, thoughts, and associations on the topic of ‘health in the workplace’. At various stages of the needs assessment different ways of participating were initiated or offered (compare also: ‘Participation’ and ‘Focus group discussion’ in chapter ‘Methods’):

- Interviews: Direct conversations on health-related challenges and resources
- Information and exchange opportunities: Regular offers on multiple channels (e.g., in-person events, emails, notices)
- Communicative validation: Feedback of results to staff in illustrated form

The participatory nature of the needs assessment - in particular by initiating discussions, encouraging questions and promoting targeted exchange - enabled the target group to actively participate in and help shape the development of the project. It became apparent that the participatory approach can not only serve to collect relevant data as part of the needs assessment, but also has the potential to increase staff interest, motivation and sense of belonging (responsibility) to the project. At the same time, trust in the research team was strengthened in the long term. In this context, trust and motivation were identified as key factors for the success of WHP programs in long-term care facilities (18). The established relationship of trust encouraged staff to openly express their individual opinions, thoughts and associations. The insights gained thus provided a realistic picture of the specific health-related needs, concerns and wishes. This can be regarded as a fundamental prerequisite for the subsequent individual adaptation of WHP interventions to the target group and the specific contextual conditions of their work environment. Future research projects should explore further possibilities for participatory involvement and participation at a higher level of participation (54) with regard to further potential and challenges.

The relationship of trust that was built up, which led to the expression of health-related needs, also created space for dilemmas to arise: With regard to the work environment, participants made various suggestions for improvements related to working conditions (see “Wishes for WHP” in the Supplementary material). But these cannot be implemented within the framework of the pilot project and could raise unrealistic expectations of the results of the WHP program (e.g., requests for more nursing staff in the pilot facility).

### Supportive management culture: the importance of company management for needs assessment in WHP programs

Leadership behavior has a strong influence on the (mental) health, well-being, and job satisfaction of employees (55–57). This connection is particularly relevant in the context of WHP, as the implementation and realization of health-promoting measures is often initiated by the company management and takes place within the existing, management-oriented corporate culture. In the preliminary project for *“HCCH,”* the willingness and commitment of company management were also identified as one of the most important factors for the success of WHP programs (18). Accordingly, the pilot facility was selected based on these aspects. The research team was able to benefit from these factors during the needs assessment: From the outset, management demonstrated

- A high degree of openness toward the project,
- A strong motivation to provide support and cooperation, and
- A fundamental willingness to allocate resources.

The *“HCCH”* pilot project showed that management motivation for the project could be maintained through regular involvement of the respective WHP initiators (e.g., exchange, consultation, information, discussions). The supportive behavior of management, which is based on interest, appreciation and transparent communication and attaches great importance to WHP, distinguishes the pilot facility. It also contributed significantly to the successful completion of the needs assessment. Despite the well-known relevance of this point for successful WHP, it is not always given in practice (56, 58).

### Focus on TCIM: use of existing TCIM-based resources and strategies for WHP

**Figure 2:**
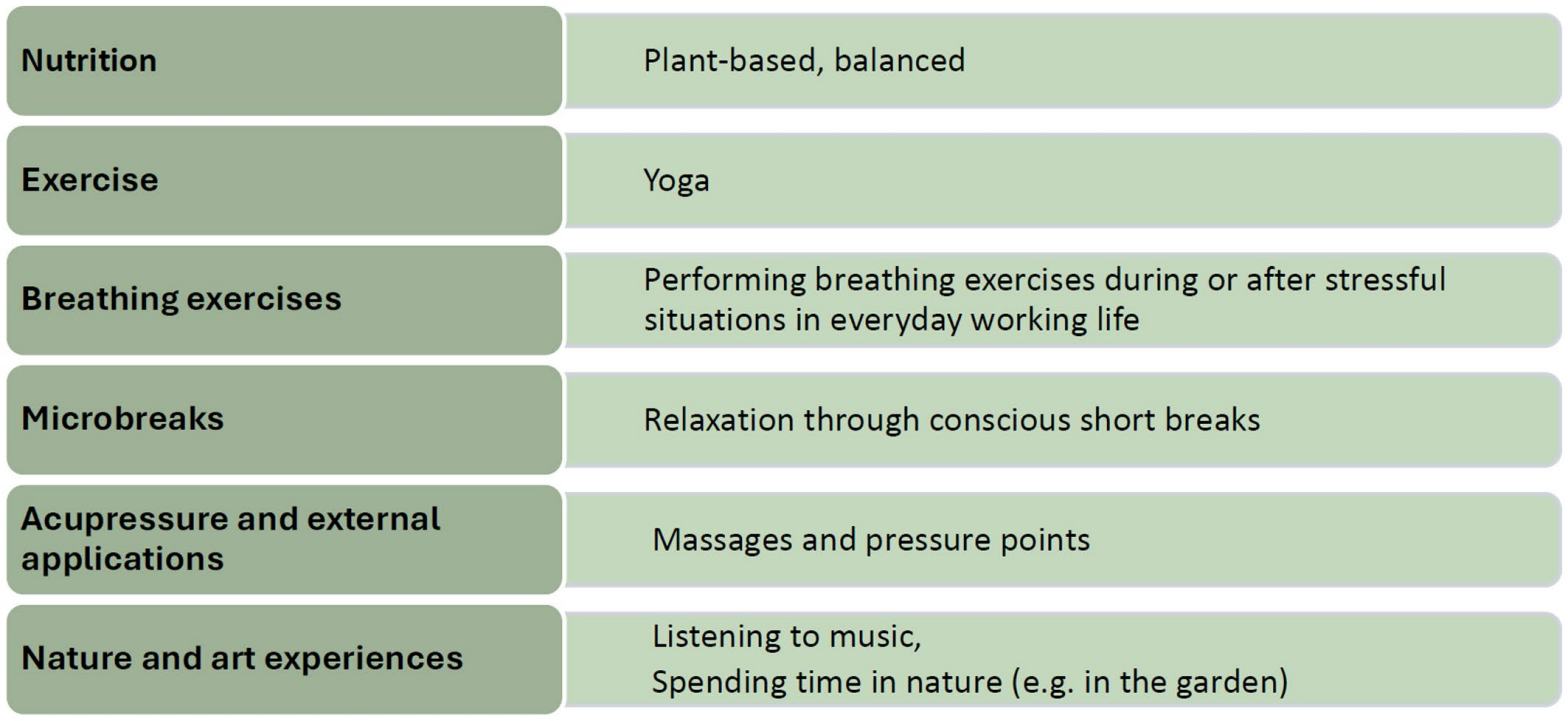
TCIM-based resources and strategies used by staff in their daily work.

The health-promoting resources and strategies identified among staff at the pilot facility that can be attributed to TCIM-based approaches are particularly relevant in the context of the planned WHP program’s explicit focus on TCIM-based measures. The results indicate that there is both a fundamental interest in and a high level of acceptance for TCIM-based interventions within the target group. Some measures, such as mindfulness exercises for oneself and others, breathing exercises, and experiences of nature and art, are already being implemented in various forms in everyday working life (see Figure 2). Against the backdrop of existing research findings on the effectiveness of TCIM-based approaches in the context of long-term care facilities, positive effects on the health and well-being of both staff and residents have been reported (23, 24). The openness of a large proportion of staff toward TCIM-based measures can therefore be considered a valuable resource. The findings open up potential for the integration of TCIM-based interventions in the WHP program. This allows measures to build upon existing behaviors and to draw on the existing knowledge and experience of staff. A key advantage of this approach is that it builds on proven and accepted behaviors and knowledge that staff already perceive as effective and helpful. This increases the likelihood that the new strategies will be successfully and sustainably integrated into everyday work. Given that the consolidation and dissemination of projects in the field of WHP are currently often inadequate (49, 50, 59–61), these results are particularly relevant.

### Relevance of TCIM-based WHP programs for the primary health care sector

In view of the increasingly challenges in the care sector - characterized by a persistent shortage of skilled workers (12), demanding working conditions (2–4) and a growing need for qualified nursing staff due to demographic change (15, 16) - promoting the health and well-being of nursing staff is becoming increasingly important. The results of this study suggest that a TCIM-based WHP program, with its natural, resource-oriented, salutogenic, and holistic characteristics (21, 62), is a promising opportunity to address these key challenges, especially in long-term care facilities. These programs are particularly suitable due to their high acceptance potential (23). It can be assumed that healthy, motivated and satisfied nursing staff are better able to provide high-quality care, adequately support residents and involve patients in preventive measures. Existing research findings suggest that promoting the health of nursing staff can improve the quality of care and patient safety (63–66) as well as patient satisfaction (67, 68).

Against this backdrop, it seems particularly relevant to consider the potential of TCIM-based WHP programs for the primary health care sector as well. Targeted promotion of the health and well-being of nursing staff in primary health care facilities - such as family doctor practices, outpatient nursing services, or community health centers (69, 70) - can make a significant contribution to relieving the burden on the health care system. It can also help ensure continuity of health care. This is achieved by keeping nursing staff, who are indispensable to primary health care, fit for work and motivated. Furthermore, the objectives of TCIM-based WHP programs - in particular prevention, health promotion and low-threshold access to health-supporting measures - are highly consistent with the central goals of primary health care (70, 71). This alignment facilitates better integration of such programs into existing primary health care structures. The conservation of resources and resulting cost efficiency of TCIM approaches can also contribute to making such programs realistic and practicable in primary health care. Through these synergies, TCIM-based WHP programs offer the potential to achieve far-reaching effects on health care professionals, patients and the health care system as a whole. They represent a strategically important approach to strengthening an effective, patient-centered and sustainable primary health care sector.

### Strengths and limitations

The methodological approach to the needs assessment follows the principles of an RPA (31, 32). This approach is designed to collect essential information about health-related factors in the respective setting within a short period of time and with the involvement of the target population.

A primary strength of the design is the combination of different data corpora. The data triangulation, recommended in the RPA, increases the validity of the results (32). This is in line with common approaches in qualitative research, where incorporating a variety of perspectives (e.g., through data triangulation) is considered a measure for improving the quality of qualitative results (35). Data triangulation also makes it possible to identify contradictions or additions by contrasting different data corpora (35), thereby introducing interesting new perspectives into research. This potential was exploited by contrasting interview and observation data (see chapter ‘Findings from the participant observations’ in the Supplementary material). This was supplemented by researcher triangulation (35) through regular discussions of the methodological approach and joint evaluation of the results. Furthermore, the results were validated by the target group itself in a moderated focus group discussion (38). The core findings from the interview and observation data were first presented to the staff of the pilot facility, and then there was an opportunity for everyone to talk about it (compare chapter “Methods”). Since no discrepancies were identified during the discussions, the reconstruction of the results by the research team can be considered sufficiently substantiated by the data material. Focusing on the results in poster format proved to be an effective way of communicating the core findings in an appealing manner that was easy for the diverse target group to understand. In addition, the illustration sparked *ad hoc* conversations and discussions among staff about health-related topics and encouraged discussions about solutions to health challenges within the team. The potential of using appealing illustrations to promote discussion on health-related topics among diverse target groups appears promising and will be further explored during the course of the project.

Thanks to the participatory elements described in the needs assessment, the research team was in close contact with the staff at the pilot facility. It is possible that the relationship of trust that developed could have influenced the results, particularly with regard to the collection and evaluation of qualitative data. The exploratory and open approach to needs assessment enabled an unbiased identification of relevant health issues from the perspective of the facility’s staff. In favor of the advantages of this approach (see chapter “Methods”), it was decided not to conduct an outcome-based, quantitative survey on classic challenges within the target group (e.g., stress, sleep disorders, burnout). The focus on qualitative data alone can be interpreted as a limitation of the study. However, a corresponding quantitative survey will be part of the planned evaluation of the WHP program and will therefore be addressed at a later date. Another limiting factor is the survey period, which was short at just under two months in the middle of summer. Collecting data over a longer timeline could provide additional insights into seasonal challenges and potentials in the work environment. The decision not to involve residents and their relatives in the needs assessment can also be seen as limiting the results, as their outside perspective on the staff could have provided additional insights into existing needs and structures.

## Conclusion

This study indicates that a quality-oriented needs assessment based on an RPA framework offers promising potentials in the context of long-term care facilities. It became apparent that the early and continuous involvement of the entire target group in the needs assessment process was well accepted in this setting. Among staff, the participatory approach fostered trust and motivation, enabling specific health-related needs to be identified realistically - a crucial basis for developing a WHP program tailored to the target group and context. The findings lead to the key recommendation for further research that a qualitative based needs assessment, oriented on a rapid participatory approach, should be established as an integral part of the preparatory measures for implementing sustainable WHP programs in long-term care facilities. A transfer to other settings is also conceivable. Furthermore, this study suggests that factors such as openness, motivation and commitment on the part of the company management contribute significantly to the successful outcome of the needs assessment. A supportive management is a key factor for success - not only in this phase, but also for the further course of the *“HCCH”* pilot project. It is therefore recommended for other researchers that the role of company management be given high priority, already in the early preparatory phases of an intervention project, such as the needs assessment. In addition, management should be regularly involved in the process - for example, through targeted exchanges, consultations, information sharing, and discussions.

The results of the needs assessment indicate that the TCIM-based approach focused on in the project is based on a fundamentally positive starting point. This applies both to the fundamental openness of staff toward appropriate TCIM-based measures and to the thematic compatibility of individual interventions with existing resources and strategies in everyday work. The extent to which this positive feedback can be confirmed and substantiated in the further course of the project remains the subject of an ongoing evaluation. If the program proves to be effective, feasible and sustainable, it can be used to develop a comparatively resource-efficient WHP program for nursing staff in long-term care settings.

Although this study focused on long-term care facilities, important conclusions can be drawn from the results for the field of primary health care. Strengthening the health and well-being of nursing staff in long-term care facilities and primary health care is not only a prerequisite for high-quality care, but also a significant contribution to the sustainability of the entire health care system. A TCIM-based approach to WHP has significant potential to help staff remain resilient and satisfied and to overcome future challenges – ultimately benefiting both caregivers and those in need of care. By promoting the health, motivation and work ability of nursing staff, such programs directly support the core values of primary health care: prevention, patient orientation, continuity and sustainability. The integration of TCIM-based WHP initiatives into primary health care settings could therefore help maintain a healthy and resilient workforce and improve the quality of care and patient safety. Ultimately, promoting the well-being of those who provide care represents a crucial investment in the resilience and long-term sustainability of primary health care systems.

## Data Availability

The qualitative data collected and analyzed in this study are not publicly available for ethical and data protection reasons, in particular to protect the privacy of the participants. For further information on the study, please contact the author responsible.

## Supplementary material

Supplementary Figure 1 Illustration key findings.
