# Supplementary material for: Qualitative participatory needs assessment in long-term care facilities: groundwork for a workplace health promotion program based on traditional, complementary and integrative medicine (TCIM)
Source: Front Med (Lausanne). 2025 Dec 5;12:1671029. doi: 10.3389/fmed.2025.1671029 (PMC12714949; doi:10.3389/fmed.2025.1671029)
Supplement: Supplementary Figure 1 — Illustration key findings. [file Image_1.pdf]

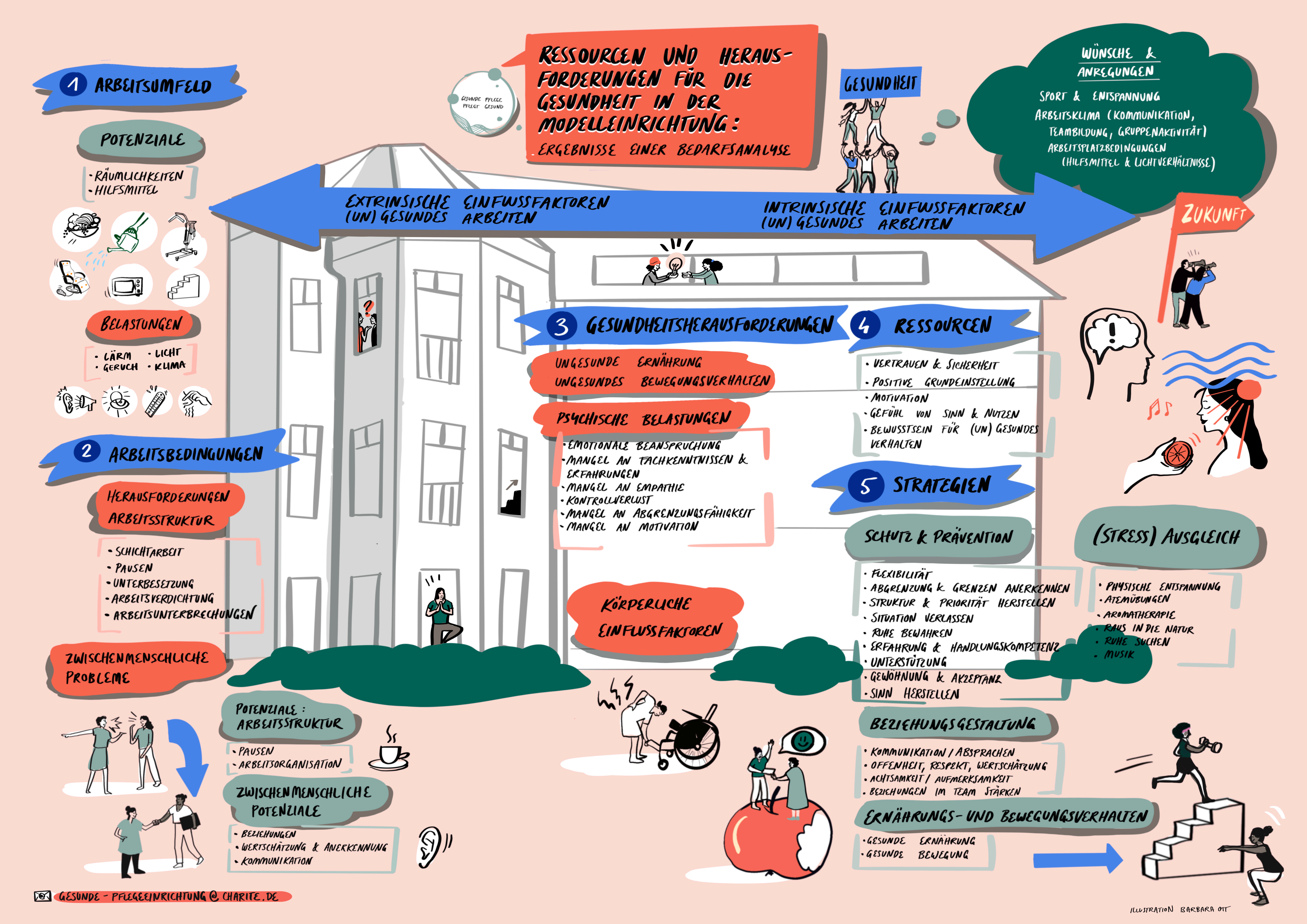

RESSOURCEN UND HERAUSFORDERUNGEN FÜR DIE GESUNDHEIT IN DER MODELLEINRICHTUNG:
ERGEBNISSE EINER BEDARFSANALYSE
GESUNDE PFLEGE PFLEGT GESUND
GESUNDHEIT
EXTRINSISCHE EINFLUSSFAKTOREN (UN) GESUNDES ARBEITEN
INTRINSISCHE EINFLUSSFAKTOREN (UN) GESUNDES ARBEITEN
WÜNSCHE & ANREGUNGEN
SPORT & ENTSPANNUNG
ARBEITSKLIMA (KOMMUNIKATION, TEAMBILDUNG, GRUPPENAKTIVITÄT)
ARBEITSPLATZBEDINGUNGEN (HILFSMITTEL & LICHTVERHÄLTNISSE)
ZUKUNFT
1 ARBEITSUMFELD
POTENZIALE
· RÄUMLICHKEITEN
· HILFSMITTEL
BELASTUNGEN
· LÄRM · LICHT
· GERUCH · KLIMA
2 ARBEITSBEDINGUNGEN
HERAUSFORDERUNGEN ARBEITSSTRUKTUR
· SCHICHTARBEIT
· PAUSEN
· UNTERBESETZUNG
· ARBEITSVERDICHTUNG
· ARBEITSUNTERBRECHUNGEN
ZWISCHENMENSCHLICHE PROBLEME
POTENZIALE: ARBEITSSTRUKTUR
· PAUSEN
· ARBEITSORGANISATION
ZWISCHENMENSCHLICHE POTENZIALE
· BEZIEHUNGEN
· WERTSCHÄTZUNG & ANERKENNUNG
· KOMMUNIKATION
3 GESUNDHEITSHERAUSFORDERUNGEN
UNGESUNDE ERNÄHRUNG
UNGESUNDES BEWEGUNGSVERHALTEN
PSYCHISCHE BELASTUNGEN
· EMOTIONALE BEANSPRUCHUNG
· MANGEL AN FACHKENNTNISSEN & ERFAHRUNGEN
· MANGEL AN EMPATHIE
· KONTROLLVERLUST
· MANGEL AN ABGRENZUNGSFÄHIGKEIT
· MANGEL AN MOTIVATION
KÖRPERLICHE EINFLUSSFAKTOREN
4 RESSOURCEN
· VERTRAUEN & SICHERHEIT
· POSITIVE GRUNDEINSTELLUNG
· MOTIVATION
· GEFÜHL VON SINN & NUTZEN
· BEWUSSTSEIN FÜR (UN) GESUNDES VERHALTEN
5 STRATEGIEN
SCHUTZ & PRÄVENTION
· FLEXIBILITÄT
· ABGRENZUNG & GRENZEN ANERKENNEN
· STRUKTUR & PRIORITÄT HERSTELLEN
· SITUATION VERLASSEN
· RUHE BEWAHREN
· ERFAHRUNG & HANDLUNGSKOMPETENZ
· UNTERSTÜTZUNG
· GEWÖHNUNG & AKZEPTANZ
· SINN HERSTELLEN
(STRESS) AUSGLEICH
· PHYSISCHE ENTSPANNUNG
· ATEMÜBUNGEN
· AROMATHERAPIE
· RAUS IN DIE NATUR
· RUHE SUCHEN
· MUSIK
BEZIEHUNGSGESTALTUNG
· KOMMUNIKATION / ABSPRACHEN
· OFFENHEIT, RESPEKT, WERTSCHÄTZUNG
· ACHTSAMKEIT / AUFMERKSAMKEIT
· BEZIEHUNGEN IM TEAM STÄRKEN
ERNÄHRUNGS- UND BEWEGUNGSVERHALTEN
· GESUNDE ERNÄHRUNG
· GESUNDE BEWEGUNG
GESUNDE-PFLEGEEINRICHTUNG@CHARITE.DE
ILLUSTRATION BARBARA OTT
