# Supplementary material for: Qualitative participatory needs assessment in long-term care facilities: groundwork for a workplace health promotion program based on traditional, complementary and integrative medicine (TCIM)
Source: Front Med (Lausanne). 2025 Dec 5;12:1671029. doi: 10.3389/fmed.2025.1671029 (PMC12714949; doi:10.3389/fmed.2025.1671029)
Supplement: Supplementary file 2 [file Data_Sheet_1.docx]

**Supplementary material: Description of results**

**Extrinsic factors: challenges and potentials in the work environment and working conditions**

The working environment at the pilot facility is perceived by staff as both health-promoting and restrictive to health. Health services and aids that promote **healthy exercise** (e.g. mobility aids, electronic beds), **healthy nutrition** (e.g. water and free fruits) or **relaxation** (e.g. massage chairs) were highlighted as positive aspects. In addition, there are certain places in the work environment (**spatial conditions**) that some people perceive as beneficial to their health, such as the garden or the break room. Some interviewees perceive existing **physical strain** as negative and harmful to their health, as illustrated by the following quote:

“*I worked upstairs [in the attic] for [...] years. So I know what it means to work up there: it's like a sauna”* (Participant_07).

Further extrinsic factors can be found in the potentials and challenges relating to staff working conditions:

Most interviewees emphasize the interpersonal potentials in the workplace, which contributes significantly to a positive working atmosphere. These potentials include **social relationships** and **good communication** within the team. Furthermore, staff often experience **appreciation and recognition** from both residents and management. In addition to the perceived potential of **breaks**, respondents rated the existing **work organization conditions** in the pilot facility as supportive and conducive to their working conditions (e.g. small and well-organized facility, proximity to company management, flexibility in shift scheduling). At the same time, staff report **interpersonal conflicts** and internal team **communication problems** due to language barriers, as illustrated by the following statement:

*“And that's just how we communicate with each other. There are a lot of people who don't speak German very well, so they have a hard time understanding each other [...]. And they don't really agree with each other. There are still a lot of conflicts.”* (Participant_02).

Many of the respondents cited various work structure conditions as causes of high stress and time pressure in their daily work, including **shift work**, **understaffing**, **frequent work interruptions**, **lack of breaks** and **increasing work intensification**. A few people mentioned that certain work organization processes and working methods of some colleagues in the pilot facility were perceived as detrimental to health (**poor work organization**), for example in relation to the economical use of resources in everyday work:

*“They don't always take the nursing trolley with them. Then you have to run around for every little thing”* (Participant_15).

**Intrinsic influencing factors: Personal health resources**

The interviews provide insight into a wide range of health resources that are considered conducive to well-being and health under the current conditions. The interviewees emphasize personal attributes that are helpful in challenging situations, in particular a **positive attitude** toward the entire work environment and a general **sense of coherence** that links their work to meaning and usefulness. Furthermore, the importance of **intrinsic motivation** is highlighted, which underlies both the pursuit of a healthy lifestyle and enjoyment of work. Many of those surveyed demonstrate a strong **awareness of health-promoting behavior with regard to nutrition and exercise**. This includes both knowledge of the potential benefits of health-promoting exercise and a balanced diet, as well as knowledge of potential risk factors in both areas. Interview participants often consider a feeling of **trust and security** in the work context to be a health-related resource.

**Intrinsic influencing factors: Personal strategies**

In addition to health-promoting resources, other intrinsic influencing factors in the form of consciously and unconsciously applied strategies were identified among the people surveyed. These help them to cope with challenging health-related situations (strategies for protection and strategies for stress management) and to strengthen existing potential (strategies for relationship building and strategies for healthy eating and exercise habits). Key aspects of protective strategies relate to **flexibility in everyday working life**, whereby certain tasks are adapted to changing situations. They also include seeking, utilizing or offering **mutual support within the team** as well as **establishing structure and priorities** for upcoming tasks. Another protective mechanism consists of becoming accustomed to and accepting certain circumstances or behaviors in the work context **(habituation and acceptance)**, for example when back pain is considered unavoidable or normal in everyday working life. Similarly, the ability to set boundaries and awareness of personal limits help to protect against influences that are harmful to health (**recognizing boundaries and limits**). **Maintaining a calm attitude** in challenging situations or deliberately **leaving challenging situations** is also an important protective strategy that enables employees to distance themselves from acute or emerging stress. There are other ways to protect oneself, including the **finding of meaning**, which endows one's actions with a coherent purpose (e.g. perceiving nursing as a calling, which engenders feelings of fulfillment and fosters personal growth). Additionally, the adoption of **rational behavior** in high-pressure circumstances is conducive to maintaining objectivity and averting impulsive reactions. **Personal experience and decision-making skills** contribute to making competent decisions and designing work processes more effectively.

Strategies for balancing stress in everyday working life help to cope with acute symptoms of stress and often manifest themselves in a **search for peace and solitude** or **spending time in nature**. Other ways to process or reduce stress include **calming breathing exercises**, **listening to music**, or performing **physical relaxation** exercises.

The interview data shows that social relationship structures in everyday working life benefit from strategies that aim to **strengthen relationship and teamwork** and take into account the rules of good **communication and coordination**. **Openness, respect and appreciation** toward colleagues has also proven helpful in shaping relationships. Some respondents value attentive and considerate behavior toward colleagues (**attention and mindfulness**), especially in situations where health issues play a role:

*“There is also the situation where you say to others: Hey, listen, when you need to go home, please go home. If you realize you can't do it, then please go home”* (Participant_04).

When it comes to their own **(healthy) nutrition and exercise habits**, many participants employ strategies to ensure that they get enough healthy exercise during the workday (e.g. taking the stairs instead of the elevator, using mobility aids). They also try to eat as balanced and healthily as possible within the work context (e.g. allowing time and space for meals).

**Intrinsic influencing factors: Personal health-related challenges**

Health-promoting resources and strategies are also confronted with various intrinsic challenges that can have a negative impact on the well-being, health, and performance of staff. The participants cited psychological stress factors as health-related challenges, ranging from **emotional strain** (e.g. when dealing with death and dying) to a feeling of **loss of control** (e.g. as a result of increasing work intensity) or **inability to set boundaries** (e.g. when colleagues' nursing practices do not meet one's own expectations). Some interviewees also express dissatisfaction resulting from the evaluation of their colleagues' work. In particular, the **lack of expertise and experience**, for example in structuring work processes, is perceived as insufficient in relation to their own requirements. In addition, the **lack of motivation**, for example to perform work tasks, and the **lack of empathy** towards human beings are also seen as shortcomings. Further health challenges became apparent through **unhealthy eating habits**, where nutrition is seen as a secondary task in the context of work: particularly during stressful periods, meals are often eaten in a hurry and while performing other activities, such as administrative tasks. **Unhealthy exercise habits** result from the demands of everyday work, such as moving heavy residents, walking long distances or sitting for long periods of time. In addition, some participants stated that personal **physical factors** and their effects are perceived as a burden (e.g. underlying physical conditions, advanced age).

**Wishes for workplace health promotion**

During the interviews, participants expressed specific wishes regarding missing WHP offerings and structures. Several interviewees pointed out the lack of **sports and relaxation offerings** that are either directly accessible at the workplace or can be easily integrated into their private lives. With regard to the **improvement of the working atmosphere**, there was a desire for targeted promotion of communication and stronger team spirit. **To optimize the nursing work structure** and conserve resources, additional support staff who could take on organizational and administrative tasks were suggested. Some participants also advocated measures to **improve the workplace conditions**, such as the purchase of air conditioning systems.

**Findings from the participant observations**

Contrasting the interview results with the participant observations largely confirms the topics identified in the interviews. However, some areas showed contrasting characteristics, as certain aspects were perceived differently in the observations than described in the interviews. The observations show that the use of “back-friendly and joint-friendly working methods” and “positioning aids and transport aids”, which were frequently mentioned in the interviews, is lacking primarily due to a lack of motivation and time pressure. A similar picture emerged regarding the existing “potentials in the work environment” (e.g. stairwell, break room) and the “strategy: Spending time in nature”. The use or application of these in everyday working life was hardly observed compared to the frequent mentions in the interviews. Further contrasts became apparent in the areas of “unhealthy eating and exercise habits”, “physical factors”, “understaffing”, “work intensification”, “work interruptions” and “lack of breaks”: all aspects are much more important than was apparent in the interviews due to their strong presence in the everyday working lives of staff.

**Findings from the focus group session**

On December 5, 2024, a 45-minute focus group session was held with some of the staff (n=16) to validate the results through communication (communicative validation). During the session, participants were first informed about the key findings of the needs assessment with the help of a poster illustration (see Supplementary Appendix 1) in order to encourage discussion (see “Focus group discussion” in the chapter “Methods”). This once again encouraged participatory involvement on the part of staff, giving participants the opportunity to share their opinions, thoughts, and associations regarding the results through discussion and exchange. The focus group meeting took place as part of a regular staff meeting. The following professional groups were present: Residential Care Assistants, Nursing Staff, Management/ Administration.

The results were accepted by the staff and thus validated through communication. In addition, during the focus group session, a) solution-oriented discussions developed on some of the results (e.g. on aids and lighting conditions) and b) ideas emerged for the constructive and health-promoting further use of the illustration. The company management decided to hang the poster in a place accessible to everyone, allowing staff to contribute their own health-promoting ideas and suggestions by sticking Post-it notes on the relevant topics on the illustration.
