# Supplementary material for: Qualitative participatory needs assessment in long-term care facilities: groundwork for a workplace health promotion program based on traditional, complementary and integrative medicine (TCIM)
Source: Front Med (Lausanne). 2025 Dec 5;12:1671029. doi: 10.3389/fmed.2025.1671029 (PMC12714949; doi:10.3389/fmed.2025.1671029)
Supplement: Supplementary file 3 [file Data_Sheet_2.docx]

**Appendix 2: Socio-demographic and professional variables of the participants**

**Table 2: Socio-demographic and professional variables of the participants (n=19)**

| Variable | Values |
| --- | --- |
| Age  Age (years), M  Age range < 50 years, n (%)  Age range > 50 years, n (%) | 52  6 (32)  13 (68) |
| Sex  Female, n (%)  Male, n (%) | 14 (74)  5 (26) |
| Occupation, n (%)  Nursing staff  Residential Care Assistant  Management/ Administration  Other | 9 (47)  4 (21)  4 (21)  2 (11) |
| Employment  Full-time (>39 hours/week), n (%)  Part-time (<30 hours per week), n (%)  Shift worker, n (%)  Professional experience (years), M | 10 (53)  9 (47)  11 (58)  18,7 |
